# Dairy Consumption and Cardiometabolic Diseases: Systematic Review and Updated Meta-Analyses of Prospective Cohort Studies

**DOI:** 10.1007/s13668-018-0253-y

**Published:** 2018-11-08

**Authors:** Sabita S. Soedamah-Muthu, Janette de Goede

**Affiliations:** 1 Center of Research on Psychology in Somatic Diseases (CORPS), Department of Medical and Clinical Psychology, Tilburg University, PO Box 90153, 5000 LE Tilburg, The Netherlands; 2 Institute for Food, Nutrition and Health, University of Reading, Reading, RG6 6AR UK; 3 Division of Human Nutrition, Wageningen University, Wageningen, The Netherlands

**Keywords:** Dairy products, Cardiometabolic, Type 2 diabetes, Coronary heart disease, Stroke

## Abstract

**Purpose of Review:**

Dairy products contain both beneficial and harmful nutrients in relation to cardiometabolic diseases. Here, we provide the latest scientific evidence regarding the relationship between dairy products and cardiometabolic diseases by reviewing the literature and updating meta-analyses of observational studies.

**Recent Findings:**

We updated our previous meta-analyses of cohort studies on type 2 diabetes, coronary heart disease (CHD), and stroke with nine studies and confirmed previous results. Total dairy and low-fat dairy (per 200 g/d) were inversely associated with a 3–4% lower risk of diabetes. Yogurt was non-linearly inversely associated with diabetes (RR = 0.86, 95% CI: 0.83–0.90 at 80 g/d). Total dairy and milk were not associated with CHD (RR~1.0). An increment of 200 g of daily milk intake was associated with an 8% lower risk of stroke.

**Summary:**

The latest scientific evidence confirmed neutral or beneficial associations between dairy products and risk of cardiometabolic diseases.

**Electronic supplementary material:**

The online version of this article (10.1007/s13668-018-0253-y) contains supplementary material, which is available to authorized users.

## Introduction

Diet-related cardiometabolic diseases such as type 2 diabetes mellitus, coronary heart disease (CHD), and stroke result in a large global health burden, accounting for over 17 million deaths in 2010 [1•]. Recent data from 195 countries showed that dietary factors have become very important risk factors contributing to worldwide deaths [2•, 3]. It is imperative that the clinical and scientific community identifies modifiable factors that can help to prevent or mitigate these cardiometabolic diseases. Specific foods and overall dietary patterns, rather than single isolated nutrients, are most relevant for chronic disease [4].

**Fig. 1:**
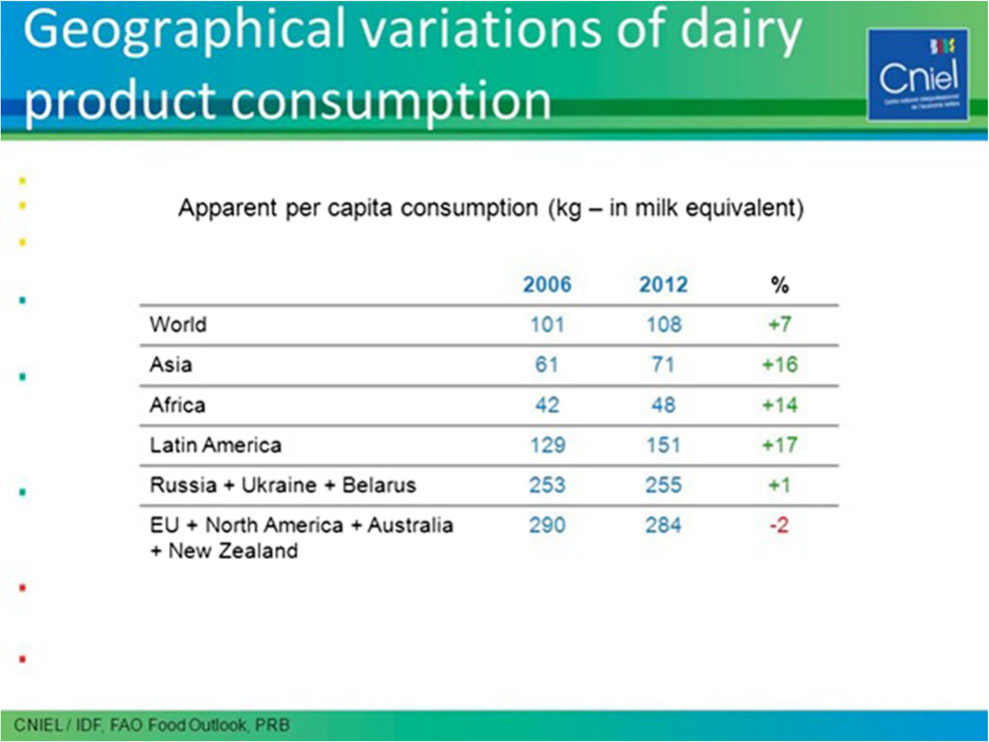
Per capita Milk Consumption from 2006 to 2012. (Available at https://slideplayer.com/slide/11662620/). From CNIEL/IDF, FAO Food Outlook, PRB [5], with kind permission

Dairy products are worldwide increasingly consumed as indicated by recent tabulations by the International Dairy Federation. From 2006 to 2013, global steep increases in per capita dairy product consumption were found. Especially, Asia, Africa, and Latin America are growing markets for dairy consumption (Fig. 1) [5].

The potential role for dairy products as part of a healthy diet was recognized by several guidelines of Western as well as Asian countries [6–9]. For example, in the US, three daily servings of dairy, mainly low-fat or fat-free, are recommended [7]. In the Netherlands, based on the 2015 food based dietary guidelines [10], daily portions of low-fat and sugar-free milk, yogurt, and cheese are recommended to fit in a balanced healthy diet [11]. The Chinese and Japanese recommendations are 300 ml of daily dairy [8] and two daily servings of milk and dairy products, respectively [9].

Dairy products such as milk, cheese, and yogurt are nutrient-dense as described in Table 1 for the main nutrient contents in these products according to the Dutch Food Composition Tables (www.rivm.nl/nevo). Of note, full-fat milk, still contains considerably less fat than low-fat cheese per 100 g product. Cheese contains 50% less water than milk and is therefore more nutrient dense than milk or yogurt. Cheese is relatively high in saturated fat, with low-fat cheese containing 8 g more fat than low-fat milk or yogurt per 100 g product. Dairy contains not only various nutrients beneficial for cardiometabolic health such as calcium, potassium, phosphorus, different vitamins such as B2, B12, D and K2, but also harmful nutrients such as sodium, saturated fat, and added sugars [12]. Vitamin D levels of dairy vary between countries depending on fortification. There has been a controversy in the literature over whether dairy products have a prominent role in healthy diets, with alternating more or less focus on beneficial or harmful nutrients [13–15].

**Table 1:** Nutrients in full, medium, and low-fat milk; cheese; and yogurt using the Dutch Food Composition Table. (Available at www.rivm.nl/nevo, accessed 12 July 2018)

		Energy (kcal)	Water (g)	Protein (g)	Carbo-hydrate (g)	Fat (g)	SAFA (g)	PUFA (g)	Ca (mg)	Na (mg)	Potas (mg)	Phos-phor (mg)	Vit B2 (mg)	Vit B12 (μg)	Vit D (μg)	Vit K2 (μg)
Milk	Full-fat	62	87.6	3.3	4.5	3.4	2.2	0.1	124	42	163	104	0.18	0.4	0	0.9
	Medium fat (semi-skimmed)	46	89.4	3.4	4.7	1.5	1	0	123	42	160	104	0.18	0.45	0	0.5
	Low-fat (skimmed)	35	90.3	3.7	4.9	0.1	0.1	0	126	44	169	106	0.18	0.44	0	0
Cheese	Full-fat 48+	369	39.3	22.9	0	30.5	19.9	0.8	816	700	86	539	0.28	2.01	0.3	63.7
	Medium fat 30+	280	45.9	30.1	0	17.7	11.5	0.5	1020	740	102	610	0.32	1.71	0.2	43.6
	Low-fat 20+	246	48.1	34.5	0	12	7.8	0.3	1059	718	112	710	0.4	2.8	0.1	32.8
Yogurt	Full-fat	58	89.2	3.8	3.4	2.9	1.9	0.1	143	42	156	114	0.16	0.24	0	0.9
	Medium fat	50	90.1	4.2	4.3	1.5	1	0	139	51	142	88	0.2	0.39	0	0.5
	Low-fat	37	91.0	4.1	4	0.2	0.1	0	152	43	159	118	0.17	0.28	0	0.1

*g* gram/100 g product, *mg* milligram/100 g product, *μg* microgram/100 g product, *SAFA* saturated fatty acids, *PUFA* polyunsaturated fatty acids, *Ca* calcium, *Na* natrium, *Potas* Potassium, *Phosphor* phosphorus

Besides high nutrient and energy densities, dairy is heterogeneous comprising many products. For example, butter is a type of dairy, yet it is often grouped with fats and oils because of the role butter has in the diet. Initially in scientific research milk and total dairy, including a variety of products, were grouped together and as such compared between studies [16–18]. In different studies, various definitions of total dairy were used, including differences in (amounts of) combined products, which makes comparisons on associations of dairy with chronic disease complex. A recent shift was made over the past 5 years, with research focusing on teasing out different dairy products by type (milk, cheese, yogurt), fermentation and fat-content (low- and full-fat dairy and milk).

Many reviews and meta-analyses summarizing associations between dairy products and cardiometabolic diseases were published in the past 20 years [17–20, 21•, 22, 23••, 24••, 25••]. Dairy products as specific foods have received increased attention, with controversies and confusion about whether or not to consume more dairy products or more specific dairy products, such as cheese, yogurt, or milk [23••, 24••, 25••, 26, 27].

This review provides the latest scientific evidence on dairy products in relation to cardiometabolic diseases (type 2 diabetes, CHD, and stroke). In addition to reviewing a large range of meta-analyses of prospective cohort studies, we identified recent cohort studies to update our previous meta-analyses on dairy in relation to type 2 diabetes, CHD, and stroke [23••, 24••, 25••].

## Methods

### Literature Search and Selection

Search syntaxes were similar to our previous meta-analyses [23••, 24••, 25••]. Published articles, without language restrictions, up to July 2018 were retrieved from PubMed complemented by hand searches of reference lists of recent reviews and meta-analyses. Eligible studies were selected using predefined criteria, i.e., prospective design and reported data on dairy consumption in relation to type 2 diabetes, CHD, and stroke. We excluded studies on animals, children aged < 18 years, and patient populations. Our previous meta-analyses were updated with nine studies [28–36]. The publication of Talaei 2017-II [36] was used to update the CHD results. The previous stroke meta-analysis already contained the (unpublished) data of that study.

### Data Extraction

We extracted descriptive study data as well as ranges of intake, medians or midpoints, numbers of subjects and stroke events, person-years at risk, and relative risks (RRs) with the corresponding 95% CIs for each category of dairy intake. Portion sizes of dairy products to convert these into grams per day, were generally reported in the included studies. If dairy intake was only reported in servings, without the actual portion size, we used portion sizes of 177 g for total, low-fat, and full-fat dairy; 244 g for total, low-fat and full-fat milk; 244 g for yogurt; and 43 g for cheese to estimate grams per day. These were mainly UK- [37] and US- [38] based portion sizes for dairy products. For open-ended upper limits of intake, we applied the same width as the adjacent category, whereas for open-ended lowest categories a zero was assigned.

### Statistical Analysis

We performed meta-analyses for type 2 diabetes on total dairy, low-fat dairy, and yogurt, for CHD on total dairy and milk, and for stroke on total dairy, milk, full- and low-fat dairy, for which the most interesting findings were reported in our previously published meta-analyses [23••, 24••, 25••]. Analyses for cheese intake and risk of stroke were not updated due to lack of new studies. If studies presented several statistical models, we included the model that included most confounders. Linearity of associations was investigated using spline analysis and dose-response meta-regression (Generalized Least-Square Trend; GLST). Splined variables were created using MKSPLINE in STATA in order to select the most appropriate knot points of nonlinear associations based on goodness-of-fit tests and Chi-square statistics. Linear and nonlinear associations were further analyzed using dose-response (GLST) meta-regression analysis. Random-effects meta-regression trend estimation of summarized dose-response data [39] was used to derive the incremental dose-response RRs. Forest plots were created for linear dose-response slopes per 200 g/d for total, low-fat, and full-fat dairy and total milk. The shape of the associations within individual studies was visualized by means of Ding’s spaghetti plots, as described previously [40]. Between-study heterogeneity was assessed by the Cochrane Q test with an I^2^ statistic [41]. Statistical analyses were performed using STATA version 11.0 (StataCorp, College Station, TX, USA).

## Results

An overview of all recent reviews and meta-analyses, and prospective cohort studies is summarized in Supplementary Table 1. In the following sections, these reviews and updated meta-analyses are presented for type 2 diabetes, CHD, and stroke.

### Dairy and Diabetes, Evidence from Prospective Cohort Studies

**Fig. 2:**
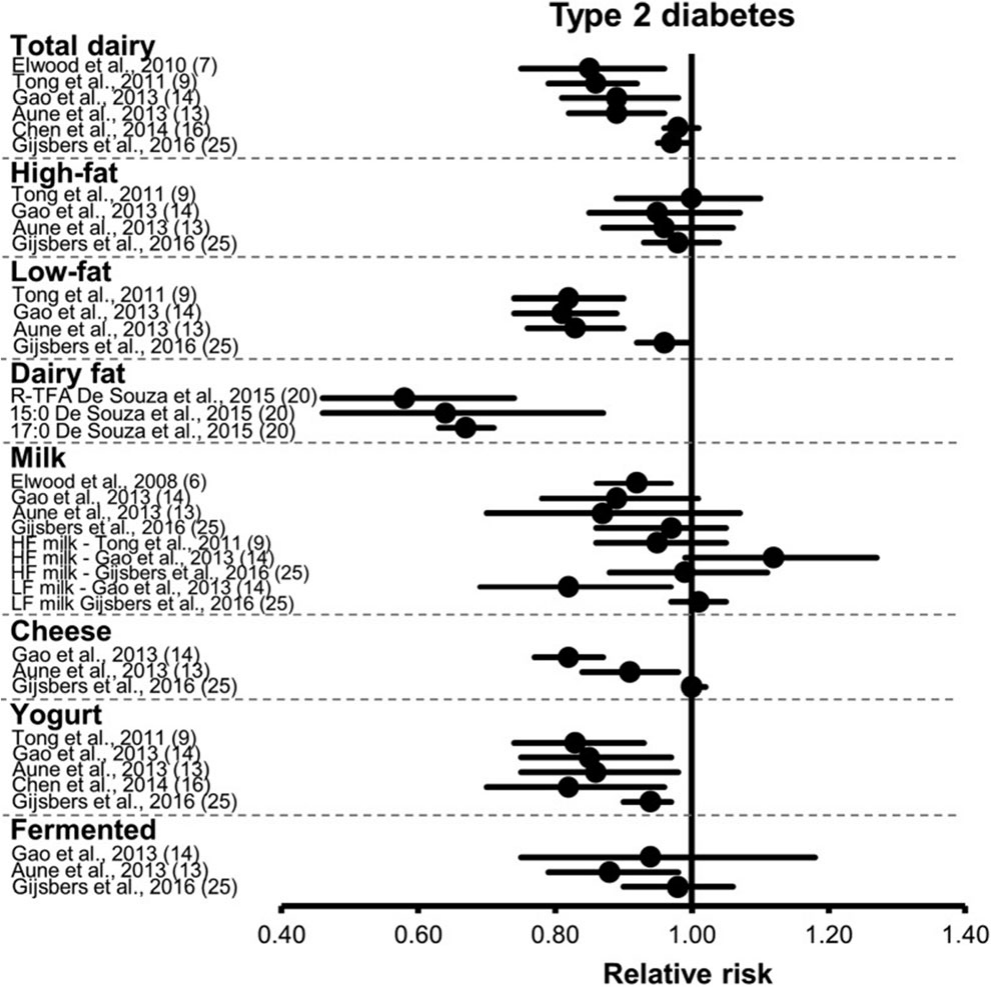
Summary of meta-analyses on dairy products and risk of type 2 diabetes by Drouin-Chartier [42••]. From Drouin-Chartier JP, Brassard D, Tessier-Grenier M, Cote JA, Labonte ME, Desroches S, et al. Systematic Review of the Association between Dairy Product Consumption and Risk of Cardiovascular-Related Clinical Outcomes. Adv Nutr. 2016;7 [6]:1026–40, by permission of Oxford University Press

The evidence on the association between dairy products and type 2 diabetes from meta-analyses was summarized recently by Drouin-Chartier et al. [42••] and Yu and Hu [43••]. Drouin-Chartier et al. [42••] summarized meta-analyses (including our work) reporting associations between various dairy products and type 2 diabetes (Fig. 2). Our work [23••] was rated as reasonably high quality (73%) compared to the other meta-analyses, according to the Meta-analysis of Observational Study in Epidemiology checklist based on the Grading of Recommendations Assessment, Development, and Evaluation scale. Comparable meta-analyses on dairy products and incident diabetes published prior to ours were from Aune et al. [44], Gao et al. [45], Tong et al. [18], Elwood et al. [17], and Chen et al. [46]. The results from all meta-analyses showed consistently neutral or inverse associations for total and low-fat dairy products, with the most striking inverse association for yogurt and type 2 diabetes. Associations for full-fat dairy, milk, and fermented dairy were consistently neutral. The results for cheese, however, differed between the meta-analyses, with an inverse association in the meta-analyses by Aune et al. [44] and Gao et al. [45], but not by Gijsbers et al. [23••], which was the most complete meta-analysis.

The meta-analysis by Aune et al. [44] included eight cheese-specific studies and the final pooled result was driven mostly by the large EPIC-Interact study [47], whereas the meta-analyses by Gijsbers [23••] included 12 cheese-specific studies (13 population samples), including large cohorts like the Nurses’ Health Study I and II, the Health Professionals Follow-up Study, the Swedish Malmo Diet and Cancer study. Gao et al. [45] did not show detailed analyses for cheese in a figure and also included fewer cohort studies.

As indicated above, dairy has been the focus in a large number of reviews and meta-analyses. Each one included a different perspective on the topic, with different numbers of studies included. Gijsbers et al. [23••] included 22 cohort studies. In a recent systematic review and meta-analysis by Schwingshackl et al. [48], 21 cohort studies on dairy intake with 44,474 type 2 diabetes cases were included, with a different selection and number of studies when compared to the meta-analysis by Gijsbers et al. Schwingshackl et al. [48] included two studies [28, 49], which were not included in Gijsbers et al., because one study [28] was published after the publication by Gijsbers et al. [23••], and one was a cross-sectional study [49]. Schwingshackl et al. [48] published more recently than our work, omitted for unknown reasons some prospective cohort studies which were included by Gijsbers et al. [50–52]. Similar inverse associations were reported by Schwingshackl et al. compared with Gijsbers et al. for total dairy. Each additional daily intake of 200 g of dairy products was inversely associated with diabetes risk (RR: 0.97; 95% Confidence Interval (CI) 0.94–0.99, I^2^ = 74%, *n* = 21 studies). In subgroup analyses, the inverse association with total dairy and diabetes was observed only in Asian and Australian studies (RR = 0.84, 95% CI 0.71–1.01), but not for American and European studies (RR = 0.98, 95% CI 0.95–1.01). This was also observed by Gijsbers et al. [23••]. Moreover, significant associations were observed by Schwingshackl et al. [48] for studies with < 1000 type 2 diabetes cases, participants ≥ 50 years of age, and a shorter follow-up (< 10 years). In subgroup analyses, low-fat dairy products showed a borderline inverse association, whereas no association could be observed for full-fat dairy products [48]. In another recent meta-analysis by Tian et al. [53], a smaller number of studies (11 cohort studies) was summarized for total dairy, full-fat milk, and yogurt results (high vs low dose), with inverse associations of 0.89 (95% CI 0.84–0.94), 0.87 (95%CI: 0.78–0.96), and 0.83 (95%CI: 0.70–0.98), respectively. These results seemed inflated compared to other meta-analyses, especially for full-fat milk, but several cohort studies were missing [46, 54, 55] and duplicate results were used [56, 57].

### Updated Meta-Analyses on Dairy and Diabetes

**Fig. 3:**
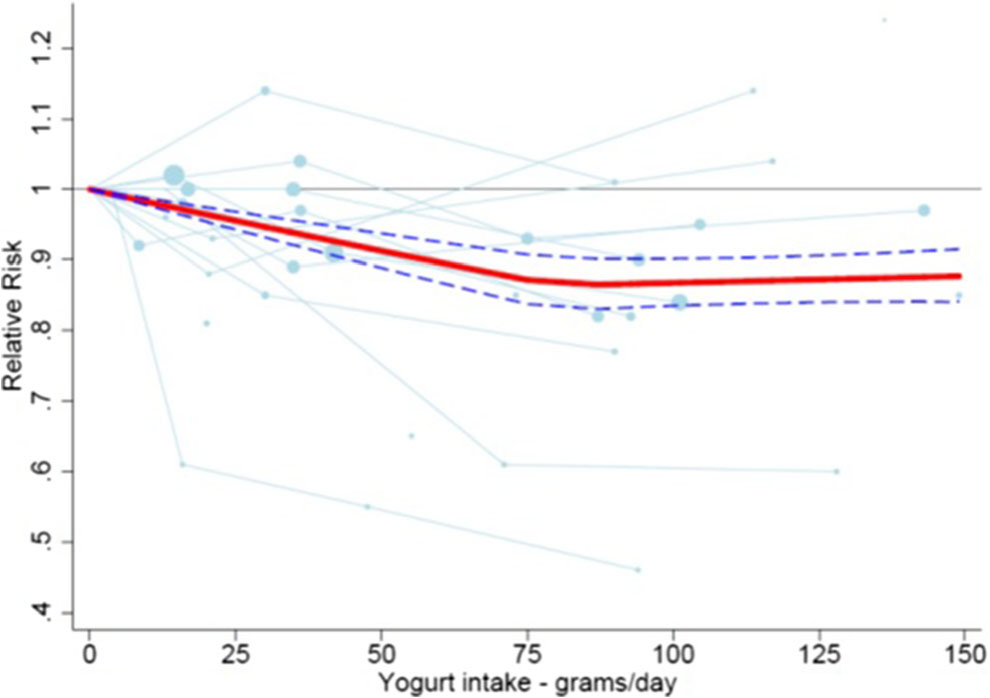
Ding’s Spaghetti plot for yogurt intake and risk of type 2 diabetes

Our previous work (Gijsbers et al.) was the most complete meta-analysis so far, and included 22 prospective cohort studies comprising 579,832 individuals and 43,118 type 2 diabetes cases [23••]. In summary, we found that total dairy was significantly linearly associated with a 3% lower risk of diabetes per 200 g/d, low-fat dairy was borderline significantly associated with a 4% lower risk per 200 g/d, and yogurt had the most striking result with a non-linear inverse significant association with diabetes (up to 15% lower risk). No associations with full-fat dairy, fermented dairy, milk, and cheese were found. In all meta-analyses by Gijsbers et al., considerable significant unexplained heterogeneity was present (*I*^2^ = 66% for total dairy, 68% for low-fat dairy, 73% for yogurt, all *p* values < 0.05). In subgroup analyses, we found a stronger inverse association for total dairy and total milk consumption in Asian populations (13–15% lower risk, although not statistically significant), compared to a null association in European populations. This may have been due to confounding adjustments (less extensive in the Asian cohorts than in the European cohorts). We updated our meta-analyses for type 2 diabetes with four cohort studies [28–30, 58] for the major findings in Gijsbers et al. [23••] and similar results were found (Table 2). In total, 26 cohort studies were included. Total dairy (per 200 g/d) was borderline significantly associated with a 3% lower risk of diabetes, and low-fat dairy was also borderline significantly associated with a 4% lower risk of diabetes (*I*^2^ = 60%) (Supplementary Fig. 1 forest plot low-fat dairy). Yogurt had the most striking result, with a non-linear inverse significant association with diabetes (RR = 0.86, 95%CI 0.83–0.90, *p* < 0.001, *I*^2^ = 69%, at 80 g/d compared with 0 g/d) (Fig. 3 Ding’s spline plot yogurt). In all these meta-analyses, considerable heterogeneity was present.

**Table 2:** New results from dose-response meta-analyses (linear and non-linear) on relationships between dairy products, type 2 diabetes, coronary heart disease, and stroke

Dairy type (increment g/d)	New studies	*N* studies (samples)	RR (95% CI)	Heterogeneity *I*^2^(%), *P*	N events; total N	Knot, *P* nonlinearity RR (95% CI) at knot
Diabetes mellitus						
Total dairy (200)	Hruby 2017 [29], Brouwer-Brolsma 2016 [28], Talaei 2018 [31], Virtanen 2017 [30]	20 (21)	0.97 (0.95–1.00)	62.8, *p* < 0.001	46,905; 5,741,718	Linear
Low-fat dairy (200)	Hruby 2017 [29], Brouwer-Brolsma 2016 [28]	15 (16)	0.96 (0.92–1.00)	60.3, *p* < 0.001	28,531; 5,313,782	Linear
Yogurt (100)	Hruby 2017 [29], Brouwer-Brolsma 2016 [28]	13 (14)	0.94 (0.91–0.97)	68.6, *p* < 0.001	37,223; 5,184,590	Non-linear 80 g/day *p* < 0.0001, 0.86 (0.83–0.90)
CHD						
Total dairy (200)	Buckland 2009 [34], Dilis 2012 [35], Talaei Singapore 2017 [36]	14 (16)	1.00 (0.98–1.03)	40.2, 0.049	11,445; 3,216,346	Linear
Milk (200)	Talaei Iran 2017 [33]	12 (13)	1.01 (0.97–1.04)	40.9, 0.061	9176; 2,231,651	Linear
Stroke						
Total dairy (200)	Haring 2015 [32]	10	0.98 (0.96–1.01)	65.6, 0.002	11,647; 2,725,832	Linear
Low-fat dairy (200)	Haring 2015 [32]	7 (9)	0.97 (0.95–0.99)	0.0, *p* = 0.68	11,092; 4,097,631	Non-linear 75 g/day, *p* = 0.007) 0.94 (0.89–1.002)
Full-fat dairy (200)	Haring 2015 [32]	6 (7)	0.96 (0.93–0.99)	0.0, *p* = 0.90	10,038; 4,076,849	Non-linear 55 g/day, *p* = 0.03 0.96 (0.90–1.01)
Milk (200)	Talaei Iran 2017 [33]	15 (17)	0.92 (0.88–0.97)	85.2, *p* < 0.001	25,377; 4,381,604	125 g/day, *p* < 0.0001 0.86 (0.83–0.89)

### Dairy and Coronary Heart Disease, Evidence from Prospective Cohort Studies

In recent years, several meta-analyses or reviews of meta-analyses have published results on dairy intake or specific types of dairy and CHD [25••, 26, 42••, 59•, 60•, 61, 62]. Drouin-Chartier et al. (2016) summarized meta-analyses reporting associations between dairy products and CHD [42••]. They concluded that, based on moderate to high-quality evidence, total dairy, full-fat dairy, low-fat dairy, milk, cheese, and yogurt consumption showed no association with the risk of CHD. Our later meta-analysis by Guo et al. [25••] was in line with those results, as well as with the meta-analysis by Bechthold et al. [59•]. Drouin-Chartier noted that associations between fermented dairy and CHD remain uncertain because of limited evidence of sufficient quality [25••, 42••]. In 2017, Gille et al. [60•] concluded that there is moderate evidence for an inverse association with CHD with the consumption of cheese based on three meta-analyses [26, 61, 62]. Guo et al. [25••] observed a moderate inverse association between fermented dairy and CVD and all-cause mortality, but not with CHD. Another recent meta-analysis with a focus only on total dairy [59•] confirmed neutral associations with CHD.

### Updated Meta-Analyses on Dairy and CHD

We updated our previous meta-analyses for total dairy and milk in relation to CHD [25••] with four cohort studies. In total, 15 cohort studies were included. Total dairy was not associated with incident CHD (Table 2). The results for milk also remained unchanged with RRs (95% CI) per 200 g/day of 1.01 (0.96–1.06) in our previous publication [25••] and 1.01 (95%CI 0.97–1.04) in the updated meta-analysis (Supplementary Fig. 2).

### Dairy and Stroke, Evidence from Prospective Cohort Studies

In recent years, several meta-analyses or reviews of meta-analyses have published results on dairy intake or specific types of dairy and incident stroke [24••, 26, 42••, 43••, 59•, 61, 63••]. In 2011, we observed a non-significant inverse association of milk with stroke risk with a RR of 0.87 (95% CI: 0.72–1.07) per 200 ml of daily intake in a meta-analysis based on six cohort studies with large heterogeneity [21•]. In a later meta-analysis by Hu et al. of dairy consumption and stroke risk, the pooled RR was 0.91 (95% CI 0.82–1.01) for high vs. low milk intake with large heterogeneity, based on nine studies. The association was nonlinear [64].

In 2016, we pooled 18 prospective cohort studies from 11 countries with 8 to 26 years of follow-up that included 762,414 individuals and almost 30,000 stroke events based on a search up to October 2015 [24••]. An increment of 200 g of daily milk intake was associated with a 7% lower risk of stroke (RR = 0.93; 95% CI 0.88–0.98; *P* = 0.004; *I*^2^ = 86%). RRs were 0.82 (95% CI 0.75–0.90) in East Asian and 0.98 (95% CI 0.95–1.01) in Western countries (median intakes 38 and 266 g/day, respectively) with less but still considerable heterogeneity within the continents. Cheese intake was marginally inversely associated with stroke risk (RR = 0.97; 95% CI 0.94–1.01 per 40 g/day). Risk reductions were maximal around 125 g/day for milk and from 25 g/day onwards for cheese. Based on a limited number of studies, full-fat milk (*n* = 4 studies) was directly associated with increased stroke risk, whereas full-fat total dairy (*n* = 6), as well as low-fat dairy (*n* = 7) was inversely associated.

The meta-analysis by Chen et al. [26], focused on cheese and no other dairy subtypes in relation to CVD, CHD, and stroke. The meta-analysis included studies until December 2015 and data largely overlapped with our meta-analysis with some minor differences: one Dutch study was missing [65] and for one other Dutch study [66] other results were used [67]. The summary RR for an increment of 40 g/d of cheese consumption was 0.94 (95% CI 0.84–1.04) for stroke risk (*I*^2^ = 64%). The largest risk reduction was observed at approximately 40 g/d. This non-linear association was also observed in our meta-analysis [24••], although in our analyses the risk reduction was maximal from 25 g per day of cheese onwards.

Drouin-Chartier et al. (2016) summarized meta-analyses (not including ours) on various dairy products and stroke [42••]. Three meta-analyses [61, 62, 64] concluded that total dairy was inversely associated with stroke, whereas we reported (nine studies) neutral associations [24••], which were confirmed by the most recent meta-analysis including 12 cohort studies by Bechthold et al. [59•] (RR per 200 g/d total dairy: 0.98, 0.96–1.00; *I*^2^: 50%; *n* = 11).

In line with our findings [24••], Drouin-Chartier et al. also found that milk and cheese were associated with a lower risk of stroke in several meta-analyses, with variations in quality of the meta-analyses [42••].

Yu and Hu [43••] presented in 2018 summaries of recent meta-analyses on dairy and dairy fat intake and cardiometabolic diseases confirming our findings on associations between dairy, cheese, milk, and yogurt with stroke incidence.

### New Cohort Studies

**Fig. 4:**
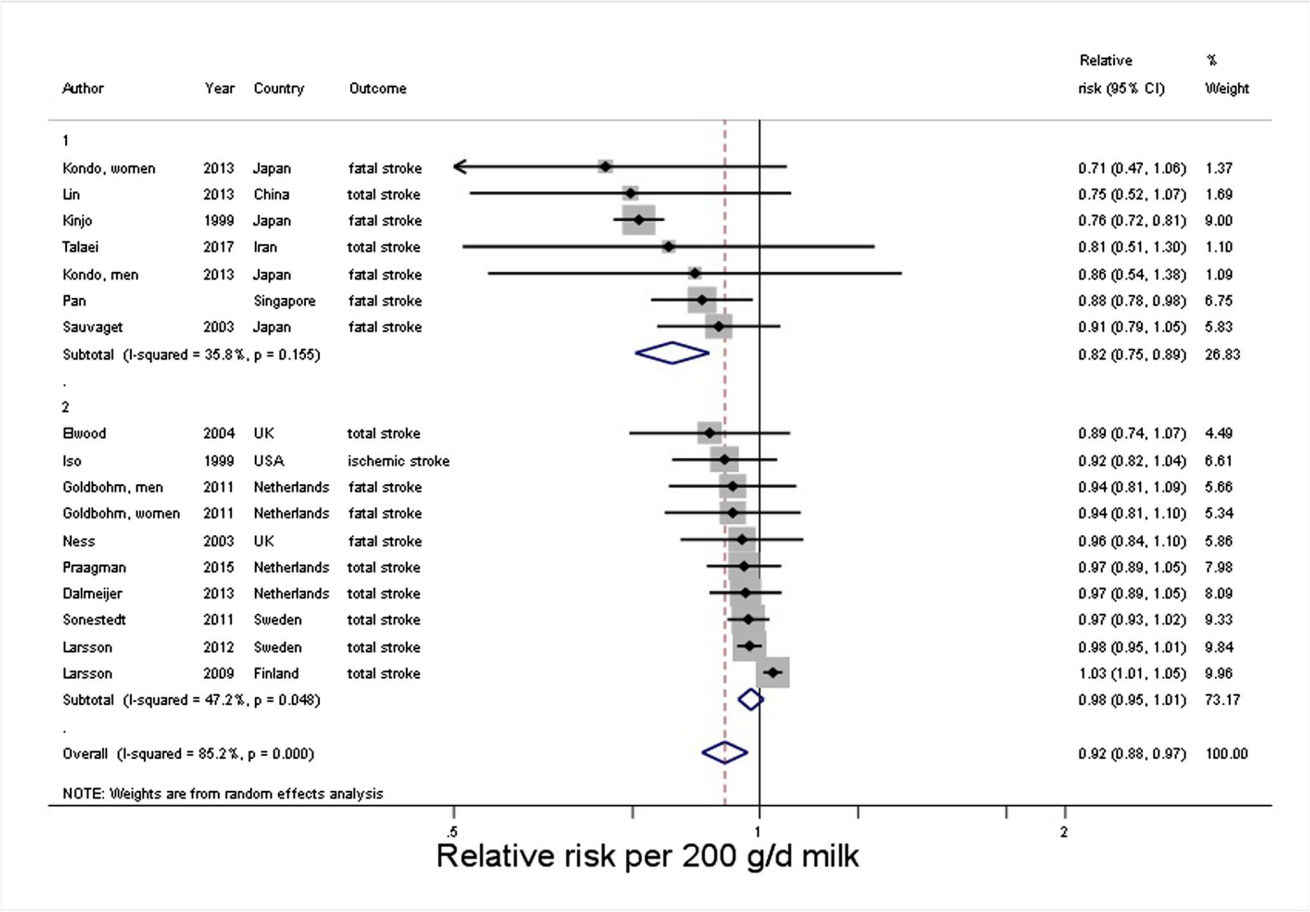
Forest plot for milk intake and risk of stroke, stratified by continent

Since our previous meta-analysis [24••], new studies have been published [32, 33, 36, 67]. We updated our previous finding by adding two new cohorts [32, 33], one with data for total, low-fat, and full-fat dairy [32] and one with data on full-fat milk [33]. Results of Praagman et al. [67] were (partly) overlapping with already included data of Dalmeijer et al. [66], which we therefore retained. The results from Talaei 2017 based on the Singapore Chinese Health Study [36] had already been included as unpublished results in our meta-analysis. Because those data were based on adjustments as requested by our group, we retained those results. For all four exposures, the results remained largely unchanged (Table 2). An increment of 200 g of daily milk intake was associated with an 8% lower risk of stroke (RR: 0.92; 95% CI 0.88–0.97; *I*^2^ = 85%). RRs were 0.82 (95% CI 0.75–0.89) in East Asian and 0.98 (95% CI 0.95–1.01) in Western countries (median intakes 38 and 266 g/day, respectively) (Fig. 4). Risk reductions were maximal around 125 g/day for milk (Supplementary Fig. 3). All milk results were similar to our previous findings [24••]. In the new studies, no new data on cheese was presented, so these results were as presented previously.

## Conclusion and Discussion

Scientific evidence from prospective cohort studies to date has suggested null or (weak) inverse associations between dairy intake and risk of type 2 diabetes, CHD, and stroke. This review, adding nine new cohort studies to existing meta-analyses, further confirmed these associations. These results should be placed in the context of observed and unexplained heterogeneity. We presented robust findings in line with previous meta-analyses [23••, 24••, 25••] and reviews.

Some differences between meta-analyses [23••, 44, 45] were reported though, even when the analyses covered the same time period of publications. These differences could be caused by different approaches (dose-response analyses versus comparing high vs. low intakes), by the categorization of studies in the various dairy type categories, or by whether or not specific studies were omitted.

Just before finalizing this review, the PURE cohort investigators published dairy data in relation to 9-year incident all-cause mortality, major CVD events, and (fatal and non-fatal) stroke in 21 countries from five continents (136,384 individuals) [68]. This international perspective is an important contribution to the field. Overall, dairy consumption was associated with a lower risk of mortality and major cardiovascular events, especially stroke, but no association was found with myocardial infarction. Milk and yogurt intake were inversely associated with all-cause mortality and major CVD events; results for milk in relation to stroke were not provided. Diabetes was not included as an outcome. Associations were consistent for regions with either low or high dairy intakes. The (strong) inverse associations of total dairy with all-cause, CVD, and stroke of the PURE study were not observed in our previous dose-response meta-analyses [24••, 25••]. It is not yet clear which types of dairy were driving the associations of the PURE study. The PURE study was still underpowered to examine the effects of specific dairy types within each region for the various outcomes, but the follow-up and population are still being expanded.

We and others described potential mechanisms previously [23••, 24••]. Regarding the association between yogurt and diabetes, probiotic bacteria, which have been reported to lower blood cholesterol, or potential effects on microbiota [69, 70] could possibly play a role. With respect to the association between milk and stroke, minerals such as calcium and potassium have been previously shown to lower blood pressure [71, 72]. Differential effects for full and low-fat yogurt or milk could not be detected.

The current discussions and confusion on health effects of fatty acids have contributed to a shift of thinking from a nutrient-based approach towards a food-based approach. Dietary guidelines traditionally mainly focused on saturated fat in dairy products and its low-density lipoprotein raising effects [6]. However, the link between food sources of saturated fat and CHD is more complicated because food sources of saturated fat contain an array of saturated and unsaturated fatty acids, each of which may differentially affect lipoprotein metabolism, as well as contribute significant amounts of other nutrients, which may alter CHD risk. Recent evidence showed more beneficial associations with cardiometabolic disease for saturated fatty acids (SFA) from dairy products than SFA from meat [73••, 74]. Furthermore, the food matrix may be important. A meta-analysis of randomized clinical trials (RCTs) studying the effect of cheese consumption on blood lipids and lipoproteins found that cheese caused lower total cholesterol, low-density lipoprotein cholesterol, and high-density lipoprotein cholesterol concentrations compared with butter, despite a similar SFA/polyunsaturated fatty acid ratio [75•].

Butter is the most SFA dense food (50% SFA). It does not contain milk fat globule membranes (MFGM), as opposed to other types of dairy. A recent RCT showed that, in contrast to milk fat without MFGM, milk fat enclosed by MFGM does not impair the lipoprotein profile [76]. A systematic review and meta-analysis by Pimpin et al. (2016) focused specifically on butter and cardiometabolic diseases [63••]. Based on four studies including 11 country-specific cohorts mainly from Europe with more than 200,000 participants and ~ 24,000 incident diabetes cases, butter was inversely associated with type 2 diabetes (RR: 0.96; 95% CI: 0.93–0.99 per daily 14-g serving). Butter was not associated with CHD or stroke. However, a recent observational study in 71,410 women, aged 50–79 years, showed that substituting butter with tub margarine (teaspoon/day) was associated with a borderline lower risk of myocardial infarction (HR = 0.95; 95% CI 0.89–1.00) after a follow-up of 13 years [77], which is consistent with what has been found for fatty acid substitution analyses [78]. The potential protective association of tub margarine may be explained by its lower proportion of SFA, trans fatty acids, and higher proportion of monounsaturated fatty acids and polyunsaturated fatty acids, compared with butter.

All evidence of dairy in relation to disease endpoints has been derived from observational studies, and residual confounding remains an issue in this type of research. There is evidence that milk and yogurt intake is related to healthy behaviors [79] and further evidence on causality is warranted. Also, the impact of dairy products per se on health cannot be fully dissociated from that of the foods it replaces [79, 80]. This needs to be investigated further in the future.

To study causality, additional evidence from RCTs and other types of designs such as Mendelian randomization studies are needed. Drouin-Chartier et al. carried out a comprehensive narrative review on the effects of dairy foods, irrespective of fat content, on cardiometabolic risk factors [81••]. That review included a range of RCTs as well as the meta-analyses of RCTs published by Benatar et al. [82] and De Goede et al. [75•], and the systematic reviews of Turner et al. [83] and Labonté et al. [84]. This paper [81••] was also cited by Gille et al. [60•] in a very recent review on fermented foods and cardiometabolic disease. RCTs, comparing high vs. low dairy or dairy vs. other foods, suggested neutral or no effects of dairy consumption on cardiometabolic risk factors, including insulin resistance, lipids, blood pressure, inflammation, and vascular function [81••]. Currently, there are no RCTs with dairy products as the main intervention with long enough follow-up periods so that cardiometabolic disease endpoints can be studied. Therefore, we are mostly relying on data from observational studies in dietary guidelines around the world. Results of short-term RCTs with effects on cardiovascular risk factors were largely consistent with large prospective cohort studies assessing associations between dairy products and cardiometabolic diseases as summarized in this review. Ideally, well-designed RCTs with long-term interventions are needed to confirm these effects on cardiometabolic disease endpoints. The design of the control group in future RCTs on dairy foods needs to be chosen carefully to ensure that foods consumed in replacement of dairy reflect consumers’ choices and dietary habits. As described by Drouin-Chartier et al. [81••] various foods used in the RCTs to substitute dairy products in the control low-dairy or dairy-free diets affect the results and interpretation and make direct comparison between RCTs difficult.

Mendelian randomization analyses have been more widely used to assess potential causal estimates of environmental risk factors with health outcomes. This type of analysis has the advantage over observational studies of minimizing confounding by using genetic markers as instrumental variables of environmental risk factors and therefore assessing causality. A recent Mendelian randomization analysis [85•] using 22 observational studies with 197,332 participants examined the causal effect of dairy consumption on systolic blood pressure and risk of hypertension, to confirm previously found inverse relationships between milk and hypertension [86]. There was no association between genetically determined dairy intake and systolic blood pressure or incident hypertension [85•, 87]. Similarly, Mendelian randomization studies found no associations between milk and diabetes [88–90] and CHD [90, 91] which is in line with intake-based results for milk in relation to diabetes and CHD. The only associations reported from Mendelian randomization studies were between genetically determined milk intake and higher BMI [92, 93]. Interestingly, no Mendelian randomization study for milk in relation to stroke has yet been performed. Lactase persistence genes used in Mendelian randomization studies characterize lactase containing dairy or milk intake, but not yogurt or cheese. Most prominent results from meta-analyses on dairy and diabetes, are found for yogurt intake and not milk or cheese. To our knowledge, it is not possible for Mendelian randomization studies to separate effects for milk, cheese, and yogurt, highlighting areas for future research.

This systematic review showed neutral or beneficial associations between specific dairy products and risk of type 2 diabetes, CHD, and stroke. In updated meta-analyses, higher milk intake was inversely associated with risk of stroke, but not with risk of CHD or type 2 diabetes. Stronger inverse associations for milk and stroke were found in Asian vs. Western populations, but this has to be investigated further with more Asian population samples. Cheese was not related to diabetes, conflicting with some of the earlier meta-analyses, depending on which studies were included. Most striking results were found for yogurt and type 2 diabetes, but this finding could also be due to more healthy behaviors which are associated with yogurt consumers [79]. Future epidemiological research should focus on more careful consideration of confounding factors, and replacement diets. Distinguishing between full and low-fat cheese and yogurt, or milk and yogurt with or without added sugars is not possible with the current literature. Additional evidence from RCTs and other study designs such as Mendelian Randomization studies should be integrated with conventional epidemiological studies to investigate further mechanisms and causality.

## Electronic Supplementary Material

Supplementary Fig. 1 Forest Plot Low-fat dairy intake and risk of type 2 diabetes (JPG 102 kb)

Supplementary Fig. 2 Forest Plot milk intake and risk of coronary heart disease (JPG 89 kb)

Supplementary Fig. 3 Ding’s Spaghetti plot for milk intake and risk of stroke. Light blue lines represent Western countries and brown lines represent Asian countries. (JPG 54 kb)

Supplementary Table 1 (DOCX 56 kb)

## Abbreviations

BMI: Body mass index
CHD: Coronary heart disease
CI: Confidence interval
GLST: Generalized least-square trend
MFGM: Milk fat globule membranes
RCT: Randomized controlled trial
RR: Relative risk
SFA: Saturated fatty acids
