# Supplementary figures and images for: Dairy Consumption and Cardiometabolic Diseases: Systematic Review and Updated Meta-Analyses of Prospective Cohort Studies
Source: Curr Nutr Rep. 2018 Nov 8;7(4):171–82. doi: 10.1007/s13668-018-0253-y (PMC6244750; doi:10.1007/s13668-018-0253-y)

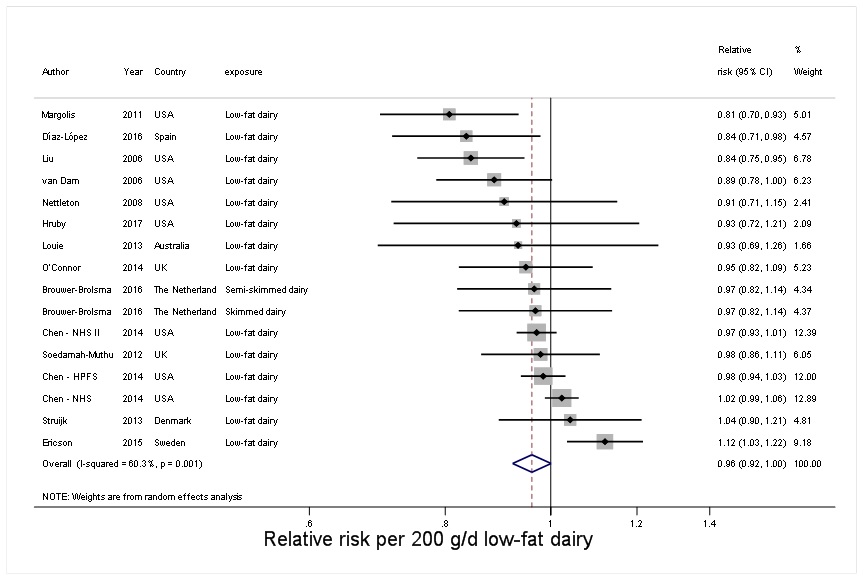

Supplement: Supplementary file 1 — Forest Plot Low-fat dairy intake and risk of type 2 diabetes (JPG 102 kb) [file 13668_2018_253_MOESM1_ESM.jpg]

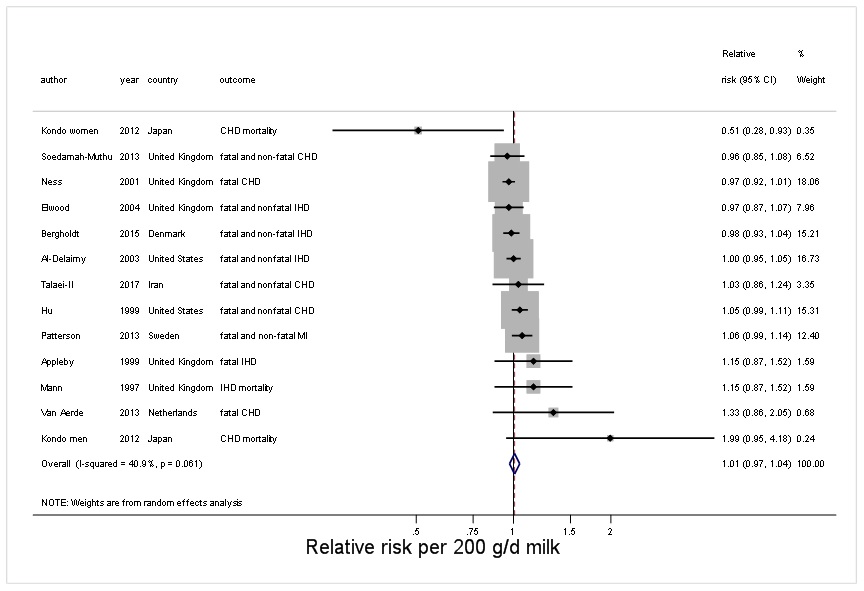

Supplement: Supplementary file 2 — Forest Plot milk intake and risk of coronary heart disease (JPG 89 kb) [file 13668_2018_253_MOESM2_ESM.jpg]

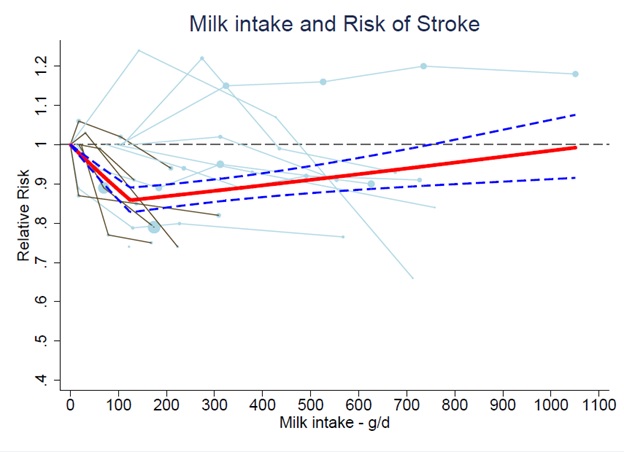

Supplement: Supplementary file 3 — Ding’s Spaghetti plot for milk intake and risk of stroke. Light blue lines represent Western countries and brown lines represent Asian countries. (JPG 54 kb) [file 13668_2018_253_MOESM3_ESM.jpg]
