# Supplementary material for: Dairy Consumption and Cardiometabolic Diseases: Systematic Review and Updated Meta-Analyses of Prospective Cohort Studies
Source: Curr Nutr Rep. 2018 Nov 8;7(4):171–82. doi: 10.1007/s13668-018-0253-y (PMC6244750; doi:10.1007/s13668-018-0253-y)
Supplement: Supplementary file 4 — (DOCX 56 kb) [file 13668_2018_253_MOESM4_ESM.docx]

Supplementary Table 1. Recent reviews and additional cohort studies for dairy in relation to type 2 diabetes, coronary heart disease, and stroke

| **Meta-analyses** | **Exposures** | **Outcomes** | **Search until** |
| --- | --- | --- | --- |
| Alexander 2016 (61) | Total dairy, ff, lf, milk, cheese, yogurt, Ca from Dairy | CVD, CHD, stroke | Studies until March 2015 |
| Aune 2014 (44) | Total dairy, ff, lf dairy, milk, ff, lf milk, fermented, cheese, yogurt, cream | T2DM | Studies until 5 June 2013 |
| Bechthold 2017 (59) | Total dairy, ff, lf dairy | CHD, stroke | Studies until March 2016 |
| Chen 2017 (26) | Cheese | CVD, CHD, stroke | Studies until Dec 2015 |
| De Goede 2016* (24) | Total dairy, ff, lf dairy, milk, low-fat milk, ff milk, cheese, yogurt, fermented dairy, butter | Stroke | Studies until Oct 2015 |
| Gao 2013 (45) | Total dairy, ff, lf dairy, milk, ff, lf milk, yogurt, cheese | T2DM | Studies until March 2013 |
| Guo 2017* (25) | Total dairy, ff, lf dairy, milk, fermented dairy, cheese, yogurt | All-cause mortality, CVD, CHD | Studies until September 2016 |
| Gijsbers 2016* (23) | Total dairy, ff, lf dairy, milk, ff, lf milk, fermented, cheese, yogurt, cream, ice-cream, sherbet | T2DM | Studies until 14 April 2015 |
| Pimpin 2016 (63) | Butter | Stroke, CHD, T2DM | Studies until May 2015 |
| Schwingshackl 2017 (48) | Total dairy, ff, lf dairy | T2DM | Studies until February 2017 |
| Tian 2017 (53) | Total dairy, ff milk, yogurt | T2DM | Studies until July 2017 |
| *from our group |  |  |  |
| **Reviews of meta-analyses** |  |  |  |
| Drouin-Chartier 2016 (42) | Total dairy, ff, lf dairy, milk, cheese, yogurt | CVD, CHD, stroke, T2DM | Studies until March 2016 |
| Gille 2018 (60) | Fermented dairy, cheese, yogurt | CVD, CHD, stroke, hypertension, metabolic syndrome, T2DM | Studies until April 2017 |
| Yu and Hu 2018 (43) | Total dairy, milk, cheese, yogurt | CVD, CHD, stroke, T2DM, hypertension, MS | Search not reported |
| **Additional cohort studies** |  |  |  |
| Brouwer-Brolsma 2016 (28) | Total dairy, ff, mf, lf dairy, fermented dairy, milk, cheese, yogurt, buttermilk, butter | T2DM |  |
| Buckland 2009 (34) | Total dairy | CHD |  |
| Dilis 2012 (35) | Total dairy | CHD |  |
| Haring 2015 (32) | Dairy,  lf dairy, ff dairy | Stroke |  |
| Hruby 2017 (29) | Total dairy, ff, lf dairy, milk, ff,lf milk, cheese, yogurt, cream and butter | T2DM |  |
| Talaei 2017 (33) Iran | Whole milk | CHD, stroke |  |
| Talaei 2017 (36) Singapore | Total dairy | CHD (*stroke) | *unpublished stroke data were already included in De Goede 2016 |
| Talaei 2018 (31) Singapore | Total dairy, milk | T2DM |  |
| Virtanen 2017 (30) | Total dairy, fermented dairy, milk, cheese | T2DM |  |

CVD=cardiovascular disease, CHD=coronary heart disease, Type 2 Diabetes Mellitus=T2DM, ff=full fat, mf=medium-fat, lf=low-fat
